# Polyimide Nanodielectrics Doped with Ultralow Content of MgO Nanoparticles for High-Temperature Energy Storage

**DOI:** 10.3390/polym14142918

**Published:** 2022-07-19

**Authors:** Ziwei Li, Hongmei Qin, Jinhui Song, Man Liu, Xiaolin Zhang, Shan Wang, Chuanxi Xiong

**Affiliations:** 1 School of Materials Science and Engineering, Wuhan University of Technology, Wuhan 430070, China; ziweili1997@163.com (Z.L.); qinhmei07@163.com (H.Q.); s18437905219@163.com (J.S.); liumanwhut@163.com (M.L.); zxl1398762480@126.com (X.Z.); 2 Hubei Engineering Research Center for Green & Precision Material Forming, Wuhan University of Technology, Wuhan 430070, China

**Keywords:** polyimide, nanocomposite films, breakdown strength, energy storage density

## Abstract

Advanced polymer dielectrics with high energy density at elevated temperatures are highly desired to meet the requirements of modern electronic and electrical systems under harsh conditions. Herein, we report a novel polyimide/magnesium oxide (PI/MgO) nanodielectric that exhibits high energy storage density (*U*_e_) and charge–discharge efficiency (*η*) along with excellent cycling stability at elevated temperatures. Benefiting from the large bandgap of MgO and the extended interchain spacing of PI, the composite films can simultaneously achieve high dielectric constant and high breakdown strength, leading to enhanced energy storage density. The nanocomposite film doped with 0.1 vol% MgO can achieve a maximum *U*_e_ of 2.6 J cm^−3^ and a *η* of 89% at 450 MV m^−1^ and 150 °C, which is three times that of the PI film under the same conditions. In addition, embedding ultralow content of inorganic fillers can avoid aggregation and facilitate its large-scale production. This work may provide a new paradigm for exploring polymer nanocomposites with excellent energy storage performance at high temperatures and under a high electric field.

## 1. Introduction

The renewable energy industries—such as electric vehicles, and wind and photovoltaic power generation—have greatly boosted the demand for advanced film capacitors that can store energy electrostatically with an ultra-high charge–discharge rate and high power density [1,2,3]. Currently, biaxially oriented polypropylene (BOPP) is the most commonly used dielectric material for film capacitors due to its extremely low dielectric loss (0.02%) and relatively high breakdown strength of about 700 MV m^−1^ [4]. However, BOPP can only operate at temperatures below 85 °C, above which the leakage current rises significantly while the breakdown strength drops sharply, resulting in a significant reduction in the capacitor’s reliability [5]. To satisfy the demands of applications in harsh conditions where the working temperature is higher than 125 °C—such as electric vehicles and aerospace power electronics—it is of significance to develop a new generation of polymer-based dielectrics for high-temperature energy storage [6].

Since polymers lose their dimensional and electromechanical stability and display large variations in their dielectric constant and dissipation factor at temperatures approaching and even higher than the glass transition temperature (*T*_g_), this has been considered a key criterion for high-temperature polymer-based dielectrics [7]. Hence, various engineering polymers with high *T*_g_ have been regarded as promising candidates for high-temperature dielectric materials [8]. However, it has been found that these high-*T*_g_ polymers typically suffer from considerable electric conduction loss under both high electric fields and high temperatures, causing remarkable temperature increases that, in turn, aggravate the conduction loss that grows exponentially with the temperature. This ultimately leads to a sharp drop in breakdown strength and, thus, a significantly reduced discharge energy density [7,9].Previous studies have shown that some commercial engineering plastics—such as aromatic Kapton PI, polyetherimide (PEI), fluorene polyester (FPE), polycarbonate (PC,) and polyethylene naphthalate (PEN)—can only discharge an energy density (*U*_e_) of 0.24, 0.89, 0.26, 0.14, and 0.26 J cm^−3^, respectively, at 150 °C when the charge–discharge efficiency (*η*) is 90%, which are far from the requirements of practical application [10].

To address the conduction loss issue of polymer dielectrics at high temperatures, plenty of strategies have been attempted in the past few decades [11,12,13,14,15]. Polymer–oxide composites are a very interesting and important research topic with various applications [16,17]. One strategy is constructing a nanoscale inorganic coating with a wide bandgap on the surface of the polymer to inhibit charge injection and, thus, reduce the leakage current [11,18]. Li et al. demonstrated that the capacitive performance of several polymer dielectrics at high temperatures—including PEI, PC, and PI—can be significantly improved by SiO_2_ coating [11]. Crosslinking is another strategy to improve the breakdown strength and the efficiency of polymer dielectrics at high temperatures [14,19,20]. Wang et al. reported crosslinked fluoropolymers exhibiting a superior *U*_e_ of 2.67 J cm^−3^ with a *η* of more than 90% at 400 MV m^−1^ and 150 °C [14]. They attributed this remarkable improvement to efficient charge-trapping by a range of the molecular trapping centers resulting from the crosslinked structures. Nevertheless, although these methods have been proven to be effective in improving the high-temperature storage performance of polymers, they are challenged by complex and rigorous manufacturing processes. In this regard, nanodielectric strategies incorporating wide-bandgap (*E*_g_) nanofillers are advantageous, since they can not only successfully inhibit the leakage current in the polymer matrix under high electric fields and elevated temperatures, but also have flexible designability and good compatibility with existing manufacturing technology [21,22,23]. To date, the two most studied nanofillers are boron nitride nanosheets (BNNSs, *E*_g_ = 5.9 eV) and alumina (Al_2_O_3_, *E*_g_ = 8.6 eV), while attempts using other wide-bandgap inorganic fillers are quite limited [24,25,26]. Since magnesium oxide (MgO) features a large bandgap of 7.8 eV and a high dielectric constant of 9.7, it is reasonable to believe that its introduction can benefit both the dielectric constant and the breakdown strength of the polymer nanodielectrics [27]. There are a lot of studies on the electrical properties of composites with nanometer MgO as fillers [28,29,30,31]. However, to the best of our knowledge, there is little research on the utilization of MgO to tune the dielectric energy storage properties of polymer nanodielectrics at high temperatures.

In this work, aromatic Kapton PI was chosen as the dielectric polymer matrix because of its excellent mechanical and electrical insulation properties, extremely high glass transition temperature of more than 360 °C, and good film-forming ability. Relying on its coordination with the carbonyl group of PI, MgO nanoparticles were directly introduced into the PI matrix, without coupling agents or surface modifiers, to prepare a series of uniformly dispersed MgO–PI nanocomposite films ranging from 0 to 0.3 vol% [32]. On the one hand, the introduction of MgO at ultralow contents can extend the interchain spacing of PI and enable easier dipole reorientation to the applied field, consequently leading to a higher dielectric constant while maintaining low dielectric loss. On the other hand, the wide-bandgap MgO improves the breakdown strength (*E*_b_) of the PI-based nanocomposite films by significantly inhibiting the leakage currents. At 150 °C, the 0.1 vol% MgO–PI nanocomposite film can discharge a maximum *U*_e_ of 2.6 J cm^−3^ and a charge–discharge efficiency of 89% at 450 MV m^−1^. Moreover, such nanocomposite dielectrics exhibit excellent fatigue resistance, and their energy storage performance does not deteriorate even after cycles at 150 °C and 200 MV m^−1^. This work may provide a new paradigm for exploring polymer nanocomposites with excellent energy storage performance under elevated temperatures and high electric fields.

## 2. Experimental

### 2.1. Materials

A polyamic acid (PAA, 18 wt%, 80~90 KDa) solution, made from pyromellitic dianhydride (PMDA) and 4,4-diaminodiphenyl ether (ODA) in N-methyl pyrrolidone (NMP), was purchased from Changzhou Furunte Plastic New Materials Co., Ltd. (Changzhou, China). MgO (~30 nm) and NMP (>99.5%) were provided by Sinopharm Group Chemical Reagent Co., Ltd. (Shanghai, China).

### 2.2. Preparation of Nanocomposite Films

MgO–PI nanocomposite films were prepared via the solution-casting method. Typically, 2.31 mg of MgO and 2 mL of NMP were first added to a beaker and, subsequently, treated for 60 min in an ultrasonic apparatus to obtain a uniformly dispersed suspension. Then, 2.5 g of PAA solution was added to the premade suspension and further magnetically stirred for 12 h to obtain a homogeneous MgO–PAA solution. After a vacuum degassing operation, the MgO–PAA solution was cast onto a clean glass plate, followed by a series of thermal treatments for imidization: 80 °C for 2 h, 100 °C for 10 min, 200 °C for 0.5 h, 250 °C for 0.5 h, 300 °C for 0.5 h, and 350 °C for 0.5 h. Finally, the 0.3 vol% MgO–PI nanocomposite film was obtained, with a thickness of about 15 μm. Accordingly, the 0.05 vol%, 0.1 vol%, and 0.2 vol% MgO–PI nanocomposite films were prepared using the same process.

### 2.3. Characterization

The cross-sectional morphology of the nanocomposite films was examined using a field-emission scanning electron microscope (Zeiss Ultra Plus, Oberkochen, Germany). X-ray diffraction (XRD) data were recorded using a D8 Advance X-ray diffractometer (Bruker AXS, Karlsruhe, Germany) with Cu Kα radiation (λ = 1.54056 Å). Fourier-transform infrared (FTIR) spectra in the range of 400–4000 cm^−1^ were recorded in the mode of attenuated total reflection (ATR) using a Nicolet 6700 spectrometer. Thermogravimetric analysis (TGA) was performed using a Netzsch instrument (STA2500, Bavaria, Germany) at a heating rate of 10 °C/min in a nitrogen atmosphere. Dielectric constant and loss tan δ as a function of frequency or temperature were acquired using an Agilent impedance analyzer (E4980A, Santa Clara, CA, USA). DC breakdown testing was performed on a Trek 30/20A high-voltage power amplifier at a voltage ramp of 500 V s^−1^. At least 15 specimens were measured for each sample, and the breakdown strength was analyzed using a two-parameter Weibull statistical method. DC conduction currents were collected at 300 MV·m^−1^ and 150 °C using a Keithley 2410 electrometer equipped with a high-voltage source. The electric displacement–electric field (*D*–*E*) loops were measured using a PolyK-CPE1901 instrument, whereby the samples were subjected to a triangular unipolar wave with a frequency of 100 Hz. Charge–discharge cycling tests were also carried out using the PolyK-CPE1901 under an electric field of 200 MV m^−1^. For all of the electrical measurements, gold electrodes with a diameter of 6 mm were sputtered on both sides of the nanocomposite films.

## 3. Results and Discussion

**Figure 1:**
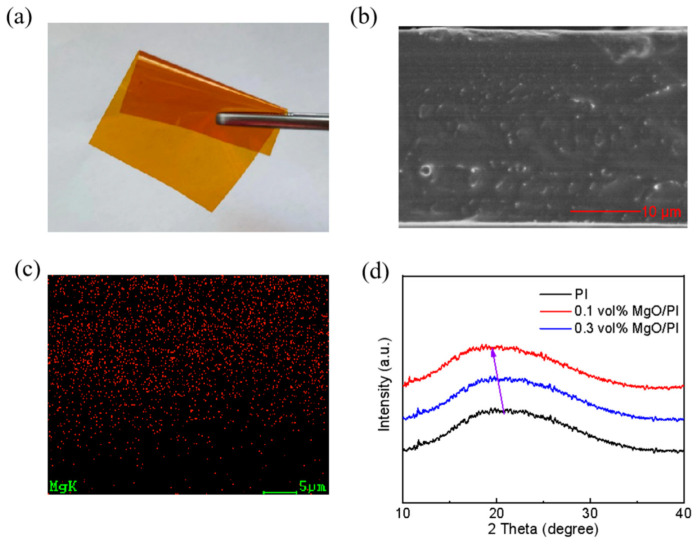
(**a**) Optical image of the 0.3 vol% MgO–PI nanocomposite film. (**b**) Cross-sectional SEM images and (**c**) EDS spectra of the 0.3 vol% MgO–PI nanocomposite film. (**d**) XRD patterns of the neat PI, and the 0.1 vol% and 0.3 vol% MgO–PI nanocomposite films.

Figure 1a shows a typical photograph of the 0.3 vol% MgO–PI nanocomposite film, demonstrating its excellent flexibility, similar to the pristine PI. Figure 1b shows a cross-sectional SEM image of the 0.3 vol% MgO–PI nanocomposite film. It can be seen that the MgO nanoparticles are uniformly distributed in the PI matrix, without obvious agglomeration, which is further confirmed by the energy-dispersive X-ray spectrum (EDS) of magnesium shown in Figure 1c. To study the possible structural changes caused by MgO nanoparticles, XRD patterns of the neat PI and MgO–PI nanocomposite films with 0.1 vol% and 0.3 vol% MgO were compared, as shown in Figure 1d. The neat PI exhibits a broad peak centering at 2θ = 20.5°, which is related to the scattering of the interchain spacing in its amorphous phase [33]. The 0.1 vol% and 0.3 vol% MgO–PI nanocomposite films also have a wide diffraction peak, with the position centering at 2θ = 19.5° and 20.1°, respectively. Since lower XRD peak position corresponds to larger interchain spacing, it is suggested that incorporation of the MgO nanofiller can enlarge the interchain spacing of PI—especially at ultralow content. This is exciting because the extended interchain spacing can enable easier dipole reorientation to the applied field, and leads to a higher dielectric constant while maintaining low dielectric loss [34].

**Figure 2:**
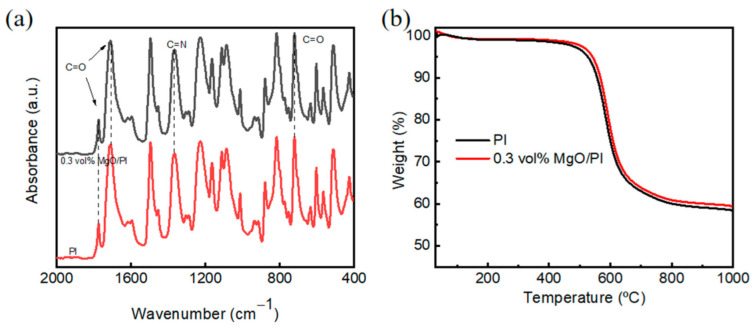
(**a**) FTIR spectra and (**b**) TGA curves of the neat PI and 0.3 vol% MgO–PI nanocomposite films.

The FTIR spectra of the neat PI and the 0.3 vol% MgO–PI nanocomposite film are shown in Figure 2a. Both spectra exhibit the typical characteristic absorption peaks of PI, involving the C-N stretching vibration at 1365 cm^−1^, along with C=O bending, symmetric stretching, and asymmetric stretching vibrations at 720 cm^−1^, 1720 cm^−1^, and 1778 cm^−1^, respectively. In addition, no characteristic absorption band of amide at 1660 cm^−1^ is observed in either sample, revealing that PAA was completely imidized to PI upon thermal treatment. The FTIR results suggest that incorporating MgO nanoparticles at this level has no adverse effect on the imidization degree of the PI matrix. This indicates that the MgO–PI nanocomposite films can maintain excellent heat resistance, similar to the neat PI. The TGA results in Figure 2b further confirm that the MgO–PI nanocomposites have a remarkable thermal decomposition temperature of more than 500 °C, similar to that of the neat PI, and this would enable them to work well under severe conditions.

**Figure 3:**
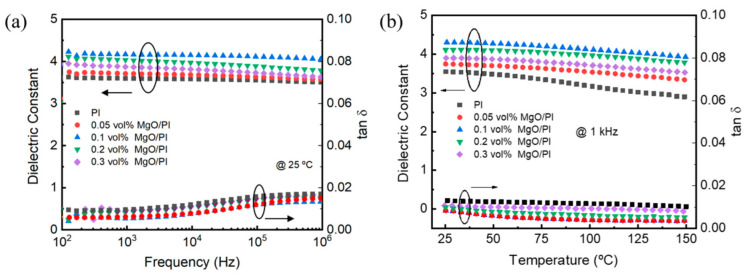
Dielectric constant and tan δ as a function of (**a**) frequency and (**b**) temperature for the neat PI and MgO–PI nanocomposites.

Figure 3a shows the dielectric constant and tan δ of the neat PI film and MgO–PI nanocomposite film as a function of frequency. Similar to the neat PI, all of the MgO–PI nanocomposite films exhibit little fluctuation in their dielectric constant over the whole frequency range of 10^2^ to 10^6^ Hz, revealing the good frequency stability of their capacitive performance. The dielectric constant of the MgO–PI nanocomposite films first increases and then decreases with the increase in the MgO content, and reaches the maximum at 0.1 vol%. Specifically, the dielectric constant of the 0.1 vol% MgO–PI nanocomposite film is 4.12 at 1 kHz, which is enhanced by about 16% relative to that of the neat PI (3.55). On the other hand, all of the MgO–PI nanocomposite films maintain a relatively low dielectric loss analogous to that of neat PI. It should be mentioned that such large permittivity enhancement by ultralow MgO content while maintaining low dielectric loss cannot be well explained by the effective medium theory alone [35]. Instead, it is more reasonable to attribute the increase in permittivity to the enlarged interchain distance, as revealed by the XRD results above. Figure 3b shows the dielectric constant and tan δ of the neat PI film and MgO–PI nanocomposite film as a function of temperature, ranging from 25 to 150 °C. The dielectric constant of the neat PI shows some decline with the increase in the temperature—especially in the range of 25 to 100 °C—probably due to the adverse effect of absorptive moisture. In comparison, the MgO–PI nanocomposite films show a relatively stable dielectric constant in the range of 25 to 150 °C, with the change being no more than 6.25%. As shown in Table 1, we compared the dielectric constant changes of MgO–PI at 25°C~150 °C with those of other, similar materials [36,37,38,39,40]. It is not difficult to find that the dielectric constant of MgO–PI changes at temperatures much lower than others. Moreover, similar to the neat PI, the MgO–PI nanocomposite films maintain low dielectric loss—less than 1%—at elevated temperatures up to 150 °C.

**Figure 4:**
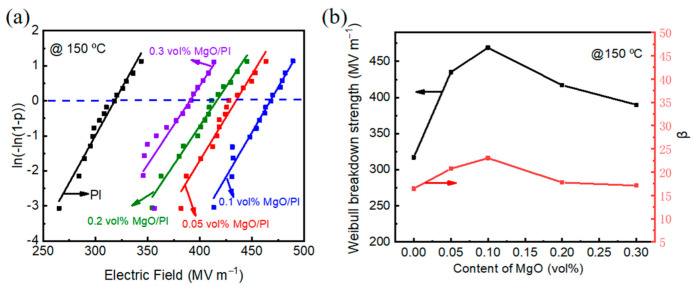
(**a**) Weibull breakdown strength of the neat PI and MgO–PI nanocomposite films at 150 °C; (**b**) Weibull breakdown strength of the MgO–PI nanocomposite films as a function of MgO content.

For a linear dielectric material, the energy storage density (*U*) can be calculated by the formula $U=\frac{1}{2}\varepsilon_{0}\varepsilon_{r}E^{2}$, where *ε*_0_ is the vacuum permittivity (8.85 × 10^−12^ F m^−1^), *ε_r_* is the relative permittivity of the dielectric material, and *E* is the breakdown strength [41]. Obviously, the energy storage density is positively correlated with the square of the material’s breakdown field strength. Since dielectric materials are inhomogeneous in reality, different internal microstructures, defects, electrode states, material sizes, and measurement conditions may cause dielectric materials to exhibit different breakdown field strengths. The breakdown data can be evaluated using the Weibull distribution, and the formula is $P\left(E\right)=1-\exp\left(-{\left(E/E_{b}\right)}^{\beta }\right)$, where *P*(*E*) is the cumulative probability of dielectric breakdown, *E* is the experimental breakdown strength, *E*_b_ is the characteristic breakdown strength when the cumulative breakdown probability is 63.2%, and *β* is the shape factor related to the sample dispersion [42]. A number of high-voltage breakdown tests were carried out, and the Weibull breakdown field strength distribution of the neat PI and MgO–PI nanocomposite films at 150 °C is shown in Figure 4a. In addition, the Weibull breakdown field strength and *β* of MgO–PI nanocomposites as a function of MgO content are plotted in Figure 4b. It can be clearly seen that the breakdown field strength of all MgO–PI nanocomposite films is higher than that of PI films. When the MgO content is 0.1 vol%, the breakdown field strengths of the composite films reach their maximum values (469 MV m^−1^), which are 1.5 times that of PI (317 MV m^−1^). Moreover, it can also be seen that the *β* value of the nanocomposite film is slightly higher than that of the neat PI film, indicating that the composite film has higher breakdown reliability. The superior breakdown strength of MgO–PI nanocomposite films may be due to MgO with a large bandgap being dispersed homogeneously in PI, effectively hindering the conduction loss. This speculation is also confirmed by the leakage current density of the MgO–PI nanocomposite films in Figure 6.

**Figure 5:**
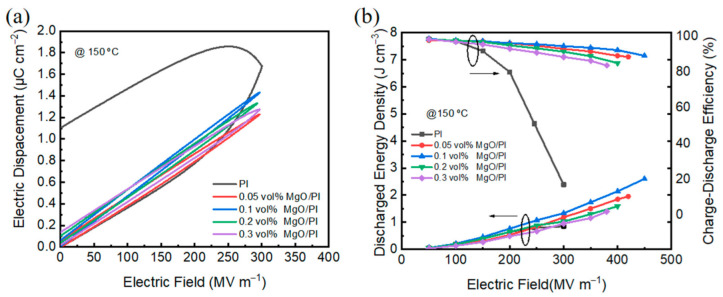
(**a**) D–E loops of the neat PI and MgO–PI nanocomposites measured at 150 °C and 300 MV m^−1^; (**b**) *U*_e_ and *η* of the MgO–PI nanocomposite films at 150 °C.

Electric displacement–electric field (D–E) loops were tested with a modified Sawyer–Tower circuit at 100 Hz and 150 °C. The discharged energy density (*U*_e_) was integrated between the discharged curve and the ordinate, and the charged density of stored energy (*U*) was integrated between the charge curve and the ordinate. Charge–discharge efficiency (*η*) is defined as the ratio of *U*_e_ and *U*. Figure 5a shows (D–E) loops of the neat PI film and the MgO–PI nanocomposites at 150 °C and 300 MV m^−1^. Apparently, the MgO–PI nanocomposite films display relatively slimmer hysteresis loops compared to the pristine PI film, indicating that the nanocomposite films deliver a lower energy loss and, thus, a higher *η*. In addition, the remanent polarization value was drastically reduced from 1.1 μC cm^−2^ for the pure PI to 0.12 μC cm^−2^ for the 0.1 vol% MgO–PI nanocomposite film, attributed to the incorporation of a slight amount of MgO, which can significantly suppress the loss of PI at high temperature. Figure 5b summarizes the discharged energy density (*U*_e_) and *η* of PI and MgO–PI nanocomposite films at 150 °C. It should be noted that the *η* of the MgO–PI nanocomposites remained greater than 80% until breakdown. Interestingly, the 0.1 vol% MgO–PI nanocomposites can even maintain a *η* of 89% at 150 °C and 450 MV/m, significantly reducing the heat accumulation caused by conduction loss, and preventing premature breakdown of the films. In addition, the *U*_e_ of 2.6 J cm^−3^ for MgO–PI nanocomposites is about three times that of the pristine PI film, delivering a *U*_e_ of 0.85 J cm^−3^ under 300 MV m^−1^ and 150 °C.

**Figure 6:**
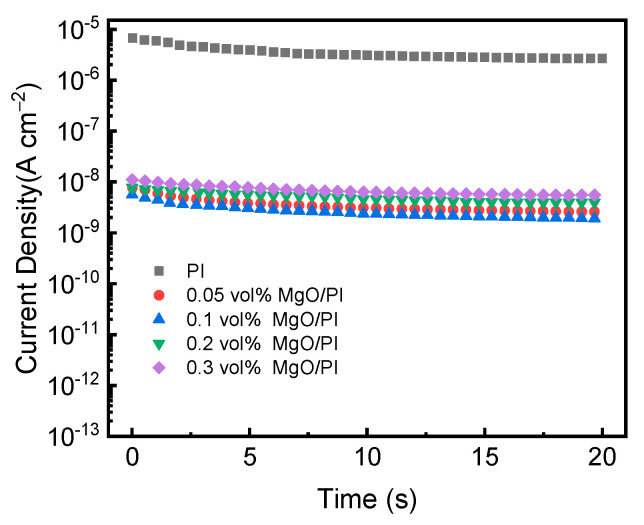
Leakage current density of the MgO–PI nanocomposite films measured under 300 MV m^−1^ at 150 °C.

To further explore the effect of MgO nanofillers on the breakdown strength and energy storage performance, the time-dependent leakage current densities of PI films and MgO–PI nanocomposite films were investigated under 300 MV m^−1^, as shown in Figure 6. It was observed that the leakage current density of the MgO–PI nanocomposites was three orders of magnitude lower than that of the neat PI; for example, that of the PI and 0.1 vol% MgO–PI nanocomposite films was 2.75 × 10^−6^ A cm^2^ and 1.90 × 10^−9^ A cm^2^, respectively. As for the MgO–PI nanocomposites films, the leakage current density increased first and then declined with the increase in MgO content. It must be acknowledged that electrical conduction loss is the major loss mechanism of dielectrics under high applied fields, so lower leakage current density of MgO–PI nanocomposites at elevated temperatures means suppressed conduction loss, which is consistent with the variation in breakdown strength shown in Figure 4 and the discharged energy density shown in Figure 5. Reduced leakage current density may have been due to the large number of deep traps provided by MgO in the nanocomposite, significantly hindering charge migration under high temperatures and electric fields [43].

**Figure 7:**
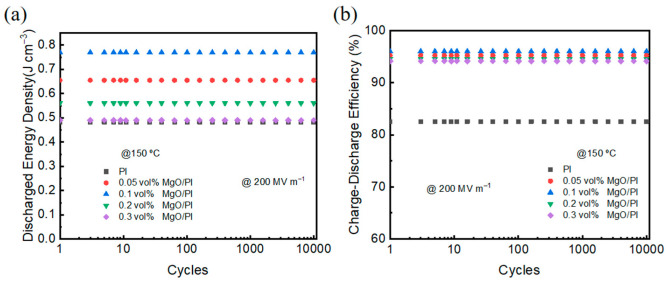
(**a**) Discharged energy density and (**b**) charge–discharge efficiency of the neat PI and MgO–PI nanocomposites as a function of cycle numbers measured at 150 °C and 200 MV m^−1^.

It is known that the continuous operation of polymer dielectrics under elevated temperatures and high electric fields may result in dielectric aging and degradation. To investigate the fatigue resistance of the MgO–PI nanodielectrics, cyclic charge–discharge tests were carried out at 200 MV m^−1^ and 150 °C, as shown in Figure 7. It is noteworthy that both the discharge energy density and the charge–discharge efficiency of the MgO–PI nanocomposite films were stable over 10,000 cycles, similar to those of the neat PI, demonstrating excellent dielectric capacitive durability.

## 4. Conclusions

In summary, a series of nanocomposite films with low volume content of nano-MgO were prepared by direct dispersion polymerization. These films have good thermal and dielectric stability, and are suitable for high-temperature applications. A small amount of magnesium oxide amplifies the molecular chain spacing of PI, and reduces the binding of dipoles, consequently increasing the dielectric constant and reducing the loss. Moreover, magnesium oxide as a filler with a wide bandgap also improves the breakdown field strength of the composite material, leading to the improvement of *U*_e_ and *η*. At 150 °C and 450 MV m^−1^, the *η* of 0.1 vol% MgO–PI is 2.6 J cm^−3^ and 89%, respectively, laying a foundation for the application of polymer-based dielectric films in the field of high-temperature energy storage.

## Figures and Tables

**Table 1:** The value of the change in permittivity of MgO–PI at 25 °C to 150 °C, in comparison with other, similar materials.

Sample	∆ε_r_/ε_r, 25 °C_
PI	8.91%
0.05 vol% MgO/PI	6.25%
0.1 vol% MgO/PI	5.71%
0.2 vol% MgO/PI	5.83%
0.3 vol% MgO/PI	6.14%
40 vol% liquid electrolyte	6.0% [36]
Bi_3_TiTaO_9_ ceramics	≈10% [37]
20 wt% BNNS/c-PS	13.3% [38]
(1 − x)AgNbO_3_-xLiTaO_3_ ceramics	≈66.70% [39]
Sr/Ba-SBN ferroelectric ceramics	>150% [40]

## Data Availability

Not applicable.
